# Supplementary material for: Palmitic Acid Promotes Lung Metastasis of Melanomas via the TLR4/TRIF-Peli1-pNF-κB Pathway
Source: Metabolites. 2022 Nov 17;12(11):1132. doi: 10.3390/metabo12111132 (PMC9696090; doi:10.3390/metabo12111132)
Supplement: Supplementary file 1 [file metabolites-12-01132-s001.zip › metabolites-2002846-supplementary.pdf]

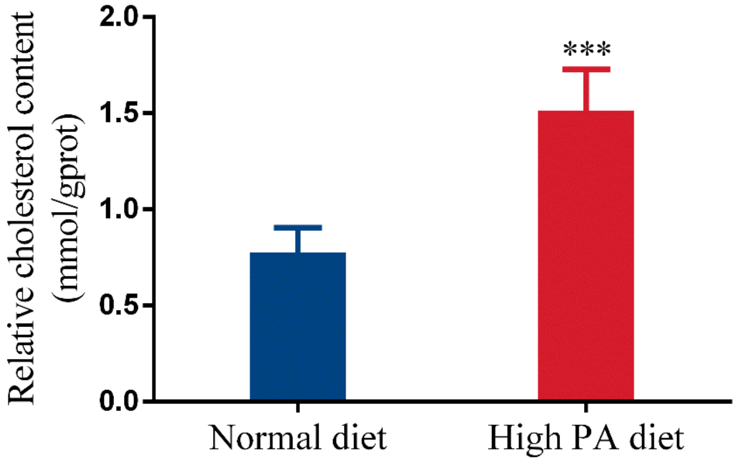

Figure S1. Serum cholesterol level

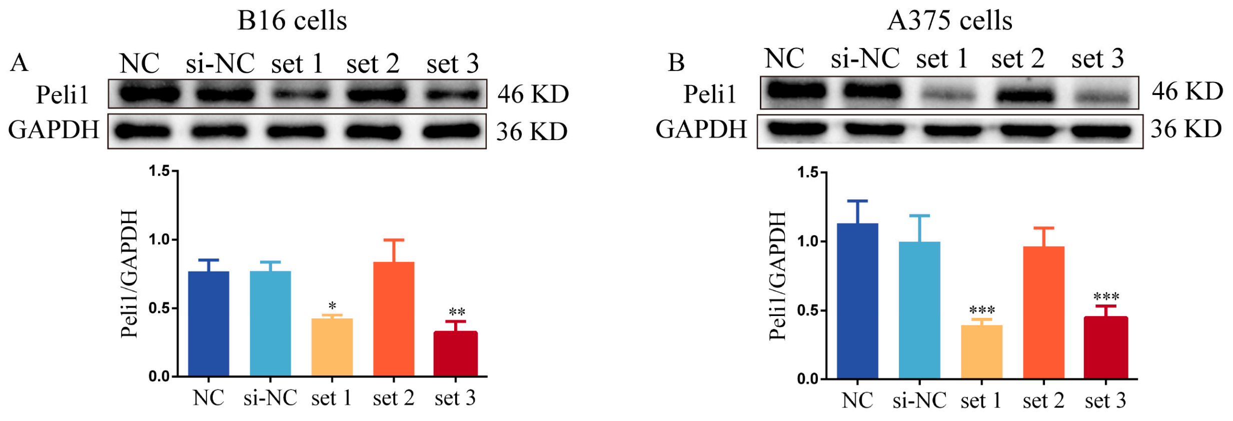

Figure S2. Interference efficiency of siRNA

(A) Interference efficiency of siRNA in B16. (B) Interference efficiency of siRNA in A375
